# Context-Dependent Role of IKKβ in Cancer

**DOI:** 10.3390/genes8120376

**Published:** 2017-12-08

**Authors:** Angustias Page, Manuel Navarro, Cristian Suárez-Cabrera, Ana Bravo, Angel Ramirez

**Affiliations:** 1 Molecular Oncology Unit, Centro de Investigaciones Energéticas, Medioambientales y Tecnológicas (CIEMAT), 28040 Madrid, Spain; a.page@ciemat.es (A.P.); manuel.navarro@ciemat.es (M.N.); 2 Oncogenomic Unit, Institute of Biomedical Investigation “12 de Octubre i+12”, 28041 Madrid, Spain; Cristian.Suarez@externos.ciemat.es; 3 Centro de Investigación Biomédica en Red de Cáncer (CIBERONC), 28029 Madrid, Spain; 4 Department of Anatomy, Animal Production and Veterinary Clinical Sciences, Faculty of Veterinary Medicine, University of Santiago de Compostela, 27002 Lugo, Spain; ana.bravo@usc.es

**Keywords:** IKKβ, cancer, animal model, transgenic mice, oncogene, tumor suppressor gene

## Abstract

Inhibitor of nuclear factor kappa-B kinase subunit beta (IKKβ) is a kinase principally known as a positive regulator of the ubiquitous transcription factor family Nuclear Factor-kappa B (NF-κB). In addition, IKKβ also phosphorylates a number of other proteins that regulate many cellular processes, from cell cycle to metabolism and differentiation. As a consequence, IKKβ affects cell physiology in a variety of ways and may promote or hamper tumoral transformation depending on hitherto unknown circumstances. In this article, we give an overview of the NF-κB-dependent and -independent functions of IKKβ. We also summarize the current knowledge about the relationship of IKKβ with cellular transformation and cancer, obtained mainly through the study of animal models with cell type-specific modifications in IKKβ expression or activity. Finally, we describe the most relevant data about IKKβ implication in cancer obtained from the analysis of the human tumoral samples gathered in The Cancer Genome Atlas (TCGA) and the Catalogue of Somatic Mutations in Cancer (COSMIC).

## 1. Introduction

Cancer is the generic name given to several dozens of different diseases with the common characteristic of lacking control of cell proliferation, leading to the production of an excessive number of cells that can affect the normal function of organs and tissues. This process is usually the consequence of one or more activating mutations in oncogenes that promote cellular proliferation and/or inactivating mutations in tumor suppressor genes. The number of genes implicated in cancer is expanding constantly as we learn more about gene function; nowadays, it is estimated that more than 1% of human genes are implicated in cancer (COSMIC v82) [1,2]). Some of these genes have the intriguing property of showing both tumor protective and tumor accelerating activities, depending on the specific mutational effect or the cellular context. One such gene is *IKBKB*, which encodes a Ser/Thr kinase named Inhibitor of nuclear factor kappa-B kinase subunit beta, which is usually abbreviated in the literature as IKK2, IKKb or IKKβ. IKKβ is a protein able to phosphorylate a number of substrates not completely known yet (for a recent review, see [3]). In the following sections, we present our current knowledge of the relationship of this multifaceted protein with tumoral transformation in different cell types.

## 2. IKKβ and the Regulation of NF-κB Pathway

IKKβ was first identified as part, along with the kinase IKKα and the regulatory subunit NF-κB essential modulator (NEMO)/IKKγ, of the IKK complex, a master regulator of the ubiquitous family of dimeric transcription factors NF-κB (for a review, see [4]). As they form a dimer, IKKα and IKKβ share common functions, mainly as NF-κB regulators but both of them also have additional individual functions not shared with the other IKK subunit [3]. NF-κB is crucial for the proper functioning of cells, as it regulates cell survival, proliferation, apoptosis and other essential processes; besides, NF-κB orchestrates both inflammation and immune responses [5,6]. Not surprisingly, considering the variety and importance of the processes regulated by NF-κB, its constitutive activity is frequently associated to cancer development [7,8]. On the other side, the inhibition of NF-κB activation decreases chemically induced lung carcinogenesis, demonstrating the contribution of NF-κB to carcinogen-induced inflammation and consequent tumor formation [9].

IκB (inhibitor of κB) is a family of inhibitory proteins of the NF-κB pathway that sequester NK-κB dimers in the cytoplasm. IκB members contain an ankyrin repeats domain, functionally implicated in the interaction with NF-κB proteins. The IKK complex is responsible for phosphorylation at specific Ser residues of members of the IκB family (for a review, see [10,11]). In general, as a result of this phosphorylation, cytoplasmic IκB proteins are polyubiquitinated and subsequently degraded by the proteasome, thus releasing NF-κB dimers and allowing their translocation into the nucleus where they can regulate gene expression. IκBα itself is a target of NF-κB, thus establishing an autoregulatory feedback loop [10,11]. The case of the IκB protein p105 (or NFκB1) is different, as it serves as both a NF-κB subunit precursor and an IκB protein. p105 is phosphorylated by IKKβ and consequently processed to an active component of the NF-κB family, p50 [12,13].

**Figure 1:**
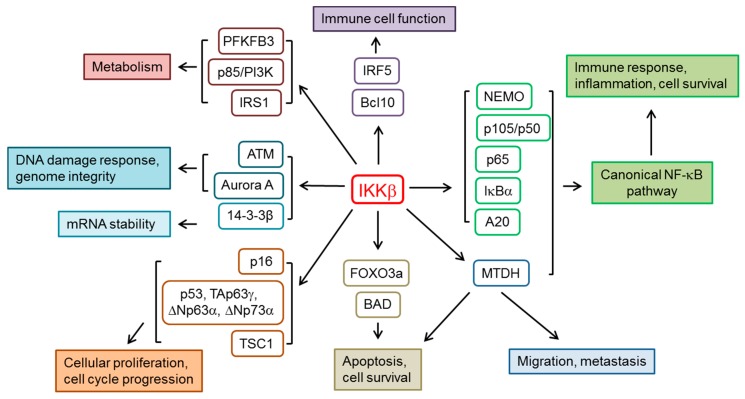
Main substrates of IKKβ and the affected biological functions. mRNA: messenger RNA. PFKFB3: 6-phosphofructo-2-kinase/fructose-2,6-biphosphatase isoform 3; p85/PI3K: p85 regulatory subunit of phosphoinositide 3-kinase; IRS1: Insulin receptor substrate 1; ATM: ataxia-telangiectasia mutated kinase; Aurora A: Aurora A kinase; TSC1: tuberous sclerosis 1; IRF5: Interferon regulatory factor 5; FOXO3a: Forkhead box O3a; MTDH: Metadherin.

The control of NF-κB activity by IKKβ also expands to other proteins different from IκB proteins (Figure 1). This is the case of the NF-κB subunit p65, whose transcriptional factor activity is increased upon phosphorylation by IKKβ [14]. Furthermore, IKKβ phosphorylates and as a result increases the stability of Tumor necrosis factor alpha-induced protein 3 (TNFAIP3, also named A20), a negative regulator of NF-κB in inflammation and immunity [15] which in turn prevents IKKβ activation [16]. NEMO, the regulatory subunit of the IKK complex, is phosphorylated by IKKβ in Ser residues at positions 43, 68 and 85; NEMO-Ser^68^ phosphorylation leads to modification of the activity of the IKK complex, which possibly represents a form of fine-tuning of NF-κB activity [17]. B-cell lymphoma/leukemia 10 (BCL10), a protein whose mutation is implicated in certain types of lymphoma, is phosphorylated at multiple sites by IKKβ and consequently degraded. BCL10 promotes NF-κB activation after receptor stimulation in several cell types, including lymphocytes. Therefore, IKKβ-mediated BCL10 phosphorylation represents a negative feedback loop for termination of NF-κB signaling in T lymphocytes [18].

In summary, IKKβ is fundamental in regulating NF-κB activity by phosphorylation of proteins with a leading role in the regulation of NF-κB activity, such as p65, IκB members and NEMO and also other NF-κB family members (A20, BCL10 or p105). By this important function, changes in the expression or activity of IKKβ can turn out in changes in the expression of many other proteins with different cellular outcomes. But, as discussed in the following section, this is not the only way that IKKβ exploits for modifying cellular proliferation and physiology.

## 3. Other IKKβ Substrates and Cellular Functions

As previously mentioned, IKKβ also phosphorylates a number of molecules that leads to changes in fundamental signaling and developmental pathways such as mechanistic target of rapamycin (mTOR), insulin and Wnt signaling. IKKβ also affects cellular metabolism, autophagy, cellular responses to DNA damage and immune responses, as discussed in the following paragraphs. Not surprisingly, changes in IKKβ activity can affect the majority of the biological capabilities considered as “hallmarks of cancer” [19]. In this section, we briefly review the most relevant non-NF-κB substrates of IKKβ, grouping them functionally.

### 3.1. Cellular Proliferation and Cell Cycle Progression

Especially important from the point of view of cancer is the activity of IKKβ over tumor suppressor proteins. p53 is one of such substrates, being phosphorylated by IKKβ at Ser 366, in the C-terminal regulatory domain. This phosphorylation marks p53 for ubiquitination and posterior degradation [20] and represents one of the reciprocal negative regulation steps that exist between p53 and NF-κB pathway [21]. IKKβ also phosphorylates other members of the p53 superfamily, as TAp63γ, hindering its interaction with the transcriptional co-activator p300 and thus inhibiting TAp63γ transcriptional activity [22]. In addition, IKKβ phosphorylates and negatively regulates ∆Np63α in response to extrinsic stimuli, as tumor necrosis factor alpha(TNFα) or chemotherapeutic agents; consequently, cells become susceptible to cell death in response to cellular stress or DNA damage [23]. Finally, IKKβ phosphorylates and stabilizes ∆Np73α in keratinocytes, thus antagonizing p53 activity; this function could be important in cellular transformation and in certain forms of cancer [24].

It is noteworthy that IKKβ can also act over p16, one of the tumor suppressor proteins encoded by the Cyclin Dependent Kinase Inhibitor 2A (*CDKN2A*) gene. In studies performed in WI-38 human lung fibroblasts and other cell lines, it was demonstrated that IKKβ binds to and phosphorylates p16 at Ser 8. This modification inactivates p16, preventing the inhibitory function of p16 over Cyclin Dependent Kinase 4 (CDK4), thus allowing progression to the phase of the cell cycle in which DNA is replicated (S phase) [25].

*TSC1* is a tumor suppressor gene whose mutation leads to the tuberous sclerosis complex syndrome, a genetic disorder that causes benign tumors in skin, brain and other organs. TSC1 is part of a protein complex that negatively regulates the activity of the mTOR pathway. It has been demonstrated in breast cancer cell lines that IKKβ is a TSC1 kinase whose activity leads to TSC1 suppression, mTOR activation and enhanced angiogenesis, favoring tumor development. Interestingly, these IKKβ-mediated alterations are also found in clinical samples of breast cancer [26].

Taken together, these data indicate that IKKβ activity could favor tumor formation by means of its negative regulation of proteins with a fundamental role in cell cycle regulation and tumor suppression, as p53 family members, p16 and TSC1.

### 3.2. Immune Cell Function

The immune system is a key element to avoid the apparition and progression of tumoral lesions. IKKβ affects the immune system by multiple ways and correct IKKβ functioning is fundamental for the establishment of a fully functional immune system, a process probably implicating both NF-κB-dependent and -independent functions of IKKβ. Thus T cell-specific IKKβ activity is needed for the formation of regulatory and memory T cells [27] and it has been recently published that genetic deletion or pharmacologic inhibition of IKKβ hampers Treg-mediated suppression of activated T cells, so improving the antitumor response of these cells [28]. IKKβ also directly regulates IRF5 (Interferon Regulatory Factor 5), a transcription factor important in the innate immune response to virus infection. IRF5 phosphorylation by IKKβ causes its dimerization and nuclear translocation, where IRF5 can activate the transcription of target genes, as interferon beta (IFNβ) and other inflammatory cytokines [29,30].

### 3.3. Metabolism

There are several links between IKKβ and metabolism. IRS1, a member of the insulin receptor substrate family, is an adaptor protein that transmits signaling from insulin and IGF1 receptors to the PI3K/AKT (Protein Kinase B) and mitogen-activated protein kinase (MAPK) pathways, among others. IRS1 mutations are associated to insulin resistance and type II diabetes. Phosphorylation of IRS1 by IKKβ can contribute to impairment of insulin signaling pathways and to the insulin resistance mediated by inflammatory processes [31].

IKKβ also influences cellular metabolism by regulating the inhibition of PI3K/AKT pathway in response to starvation. This effect is mediated by phosphorylation of the p85 regulatory subunit of PI3K in response to nutrient deprivation. Interestingly, these results have been observed both in cultured cells and in a variety of tissues in response to metabolic restriction [32]. Another mechanism of modulation of cellular energy metabolism by IKKβ comes from the regulation of 6-phosphofructo-2-kinase/fructose-2,6-biphosphatase isoform 3 (PFKFB3), a major driver of aerobic glycolysis. IKKβ phosphorylates PFKFB3, leading to its subsequent inhibition in the low glutamine environments that can take place as tumors grow. This mechanism is considered an adaptation to metabolic stress, increasing the ability of cancer cells to survive at low glutamine concentration [33].

### 3.4. DNA Damage, Genome Integrity and mRNA Stability

Currently, we know a number of additional IKKβ substrates that take part in fundamental cellular processes and whose deregulation is related to some forms of cancer (for a review, see [3,34,35]). Among them, we can cite ATM, a protein kinase implicated in the response to DNA damage that is mutated in the disorder ataxia-telangiectasia. In case of DNA damage, IKKβ is activated, translocates into the nucleus and phosphorylates ATM, promoting DNA repair [36].

Also important from the point of view of cancer is the role that IKKβ plays in the regulation of Aurora A kinase, an important regulator of cell cycle progression and maintenance of spindle bipolarity. Reduction of IKKβ activity leads to an increase in the amount of Aurora A and concomitantly to spindle defects, aneuploidy and cellular transformation [37]. Recently, Shen et al. have described that decreased phosphorylation of Aurora A by IKKβ leads to cell division and developmental defects in a zebrafish model of embryos lacking IKKβ [38].

Finally, phosphorylation of 14-3-3β by IKKβ results in modulation of posttranscriptional regulation of gene expression by affecting messenger RNA (mRNA) stability [39] and could therefore lead to changes in multiple cellular functions related to cancer.

### 3.5. Apoptosis, Cell Survival and Cell Migration

FOXO3a is a transcription factor that controls cell cycle progression and induces apoptosis. In some tumors, as a consequence of AKT activation, FOXO3a is excluded from the nucleus, thus being unable to regulate the transcription of its target genes and contributing to tumor development. Hu et al. have found that several breast tumors have nuclear exclusion of FOXO3a not in response to AKT activation but associated to expression of IKKβ: so, IKKβ inactivates and directs FOXO3a to degradation, thus inducing tumorigenesis in breast cells [40].

Interestingly, the study of the TNFα-induced phosphoproteome in the MCF-7 breast cancer cell line has revealed MTDH as other IKKβ substrate [41]. MTDH is a regulator of multiple pathways, including Wnt and NF-κB (through IkBα degradation). MTDH acts as an oncogene and promotes cell proliferation and migration, metastasis and angiogenesis; in addition, its overexpression correlates with bad prognosis in several cancer types, including melanomas, breast tumors, hepatocellular carcinomas and esophageal squamous cell carcinomas (SCCs). MTDH phosphorylation by IKKβ is important for TNF-α-mediated gene expression and NF-κB regulation [41]. In other contexts, IKKβ can also inhibit TNFα-induced apoptosis by phosphorylation-mediated inhibition of the proapoptotic protein BAD [42].

## 4. Mouse Models for the Study of IKKβ Role in Cancer

Experiments performed almost 20 years ago revealed that IKKβ functions cannot be assumed by IKKα or other proteins, as *Ikbkb* knock-out mouse embryos die at mid-gestation due to massive apoptosis in the hepatocytes [43,44,45], a phenotype similar to that observed in knock-out mice lacking p65 or other NF-kB subunits. The lethal phenotype of IKKβ-null embryos is partially rescued by simultaneous inactivation of the receptor 1 of tumor necrosis factor (TNFR1), indicating that the hepatic apoptosis observed in IKKβ-null mice is dependent on TNFα toxicity [45]. Curiously, the consequences of a lack of IKKβ in humans seem to be less harmful as homozygous deletion of the *IKBKB* gene is not embryonic lethal—at least in some patients—but leads to a lack of Treg and γδ T cells and to severe immunodeficiency [46].

In the last years, animal models with tissue-specific modified IKKβ expression have shed light on the divergent functions of IKKβ in tumoral transformation of different organs and cell types (lung, melanocytes, pancreas, liver, intestine, skin and oral stratified epithelia), as we describe in this section (summarized in Table 1).

### 4.1. Lung Cancer

In lung, IKKβ activity in alveolar epithelial cells contributes to tumoral transformation and cancer development, as its genetic inactivation impairs tumor proliferation induced by concomitant Kras^G12D^ and p53 knock-down and increases tumor latency [47]. In addition, IKKβ chemical inhibition in mouse models of lung cancer also reduces tumor cell proliferation and tumor growth [47,48] and slows tumor progression [48]. Furthermore, Zaynagetdinov et al. showed that transgenic mice with inducible expression of active IKKβ in airway epithelial cells, following exposure to chemical carcinogens, exhibit enhanced lung tumorigenesis. Increased tumor formation was preceded by increased proliferation of airway epithelial cells and enhanced influx of regulatory T lymphocytes [49].

Interestingly, IKKβ activity in myeloid cells also affects tumorigenesis, as IKKβ ablation in this cell population decreases the pulmonary inflammatory process induced by tobacco smoke and abrogates lung tumorigenesis [50].

### 4.2. Melanoma

Mice with melanocyte-specific *Ikbkb* deletion showed less melanoma incidence than *Ikbkb* wild type mice in a melanoma model that lacks p16 and p19 and expresses a mutant form of H-Ras. This melanoma-promoting activity of IKKβ is mediated, at least partially, by preventing cell cycle arrest and through modification of the expression of regulators of cell cycle, as CDK2, CDK4 and Aurora kinases A and B [51]. These authors also studied the effect of IKKβ in myeloid cells during melanoma tumorigenesis in a series of experiments injecting intravenously allogenic Braf^V600E^/Pten^−/−^ or syngeneic B16F0 melanoma cells into recipient mice lacking IKKβ in myeloid cells. At difference to the effect described above for lung cancer, IKKβ in myeloid cells is essential for the establishment of an efficient antitumorigenic immune response: myeloid cells lacking IKKβ are less able to phagocytize and digest melanoma cells than myeloid cells expressing wild type IKKβ [52]. These results reveal an interesting aspect of the role of IKKβ in cancer: IKKβ actually has both tumor promoting and tumor suppressing activities over melanoma development, depending on the targeted cell type (melanocytes and myeloid cells, respectively).

### 4.3. Pancreatic Cancer

Constitutive IKKβ activity promotes leukocyte infiltration and induces acute pancreatitis [69,70]. Pancreas-specific genetic deletion of *Ikbkb* in a Kras^G12D^ mouse model of pancreatic ductal adenocarcinoma considerably delayed carcinogenesis and led to lower grade pancreatic lesions. These effects depend partially on modifications of IL-1α induction and of NF-κB activity on the one hand and on downregulation of Notch signaling and of the expression of the Notch-target genes *Hes1* and *Hey1*, on the other hand [53,54].

### 4.4. Liver Cancer

In hepatocellular carcinoma (HCC) studies, a somewhat more complex situation has been described: in an experimental model of hepatic carcinogenesis induced by diethylnitrosamine (DEN), a lack of IKKβ in hepatocytes results in an increase in reactive oxygen species (ROS) production and in the incidence of HCCs—both in number and size—indicating an antitumoral role for IKKβ in hepatocytes. By contrast, deletion of IKKβ in both hepatocytes and Kupffer cells (liver resident macrophages which produce mitogens in response to liver damage) led to the development of fewer HCCs than mice expressing normal amounts of IKKβ, indicating a IKKβ protumoral role in Kupffer cells [55]. The effect of hepatocyte IKKβ over HCC is mediated by negative regulation of signal transducer and activator of transcription 3 (STAT3) signaling [57] and by c-Jun N-terminal kinases (JNK) pathway [56].

### 4.5. Intestinal Cancer

Regarding intestinal cancer, different animal models have been studied. *Ikbkb* deletion in intestinal epithelial cells by a Villin-Cre transgene resulted in lower colon cancer incidence in a carcinogen-induced model, by enhanced apoptosis during tumor promotion [58]. *Ikbkb* deletion in myeloid cells by a Cre transgene directed by regulatory elements from the Lysozyme 2 gene had also an antitumoral effect but mediated by a decrease in expression of proinflammatory mediators [58]. The expression of a constitutively active form of IKKβ in intestinal epithelial cells of transgenic mice induced spontaneous intestinal tumor formation, as well as enhanced tumorigenesis in models of carcinogen- or mutation-induced colorectal cancer [59]. These effects were accompanied of increased production of cytokines and chemokines and of increased β-catenin activation and Wnt signaling in the intestinal inflammatory microenvironment [59]. The mechanism through which IKKβ activation in intestinal epithelial cells accelerates intestinal tumor promotion is the upregulation of inducible nitric oxide synthase (iNOS), which induces DNA damage [71].

Recently, two reports about the role of IKKβ in mesenchymal cells over intestinal tumorigenesis have been published [60,61]. These articles are a good example of how very different outcomes can be obtained in response to subtle differences in the induced IKKβ alterations, illustrating the complexity of IKKβ functions. Constitutive *Ikbkb* deletion in mesenchymal cells by means of a ColVI-Cre transgene led to protection against inflammation-induced intestinal carcinogenesis [60], indicating an intestinal tumor-promoting role for IKKβ in mesenchymal cells. When mesenchymal *Ikbkb* inactivation was accomplished by an inducible Col1a2Cre-ER transgene, intestinal tumor growth is promoted upon carcinogenic treatments [61]. As the authors of these reports discuss, these apparently opposite results could be due to differences in the mesenchymal populations targeted in both experiments, being the Col1a2Cre-ER transgene expressed in more cells that the ColVI-Cre transgene. Other possible explanation relies on the temporal differences in *Ikbkb* inactivation in both models. In addition, IKKβ could have distinct functions in different fibroblast populations.

### 4.6. Non-Melanoma Skin Cancer, Oral and Esophageal Cancer

Mice with *Ikbkb* inactivation in skin keratinocytes suffer from a TNFα-mediated inflammatory skin disease that is rescued in the absence of TNFR1 [62]; these results highlight the importance of keratinocyte IKKβ in the maintenance of a correct balance of inflammatory cells in the skin. Interestingly, *Ikbkb* deletion in skin resulted in epidermal hyperplasia and activation of STAT3 and ERK1/2 pathways but not in tumor formation [63]. Other animal models with floxed *Ikbkb* genes aimed to study IKKβ functions in T cells and astrocytes led unexpectedly to cre-mediated *Ikbkb* deletion in skin keratinocytes. These mice showed a hyperplasic skin phenotype associated to inflammation that in some cases led to SCC development [64,65].

The expression of a constitutively active form of IKKβ in epithelial cells of murine esophagus leads to esophagitis and increased angiogenesis in the esophageal stroma. These phenotypes were accompanied by increased esophageal infiltration of immune cells and increased levels of GM-CSF and TNF [68]. Unfortunately, the reduced life span of these mice makes difficult to perform experiments for the study of the contribution of IKKβ to esophageal carcinogenesis.

We have studied mice overexpressing human IKKβ in stratified epithelia under the transcriptional control of Keratin K5-derived regulatory sequences; this model is also illustrative of the varying effects of IKKβ over different cell types. K5-IKKβ transgenic mice are prone to the appearance of oral epithelia lesions in palate and forestomach, associated to a higher presence of infiltrating cells. These spontaneous lesions were frequently benign (from hyperplastic dysplasia to carcinoma in situ) but SCCs were also observed in some animals [67]. When K5-IKKβ is accompanied by a v-Ha-Ras transgene (Tg.AC mice [72]) and subjected to an experimental oral cancer treatment, control Tg.AC mice developed benign oral lesions, whereas the majority of double transgenic K5-IKKβ/Tg.AC mice developed multiple foci of highly dysplastic invasive SCCs in the oral epithelia and in the non-glandular stomach. In short, overexpression of IKKβ in murine oral epithelia leads to an increase both in spontaneous tumorigenesis and in the malignancy observed after chemical induction of oral tumors, indicating an oncogenic role for IKKβ in oral cancer [67].

However, when we probed this transgenic line for the role of IKKβ in non-melanoma skin cancer, a completely opposing outcome was obtained, as mice overexpressing IKKβ in keratinocytes were refractory to development of skin tumors under carcinogenesis protocols, indicating a tumor suppressor role for IKKβ in skin [66]. K5-IKKβ keratinocytes express an increased amount of several tumor suppressor proteins (p53, p16 and p19). In order to check if the antitumoral effect of IKKβ in skin is related to any of these tumor suppressor proteins, we performed carcinogenesis experiments in K5-IKKβ mice that simultaneously lack p53 in skin or p16 and p19 in every cell. These experiments showed that the skin tumor protective function of IKKβ is independent of p53 but dependent on the proteins coded by the *Ink4a/Arf* locus (p16 and p19), as the number of tumors generated in K5-IKKβ mice in the absence of p16 and p19 were similar to the number generated in control mice; interestingly, in *Ink4a/Arf* null background, K5-IKKβ mice developed a significantly higher amount of undifferentiated and spindle SCCs (the most malignant types of SCCs), indicating that the skin tumor suppressive function of IKKβ turns into an oncogenic role in absence of p16 and/or p19. In order to clarify which *Ink4a/Arf*-coded tumor suppressor protein mediates these IKKβ skin suppressive cancer functions, it will be needed to generate K5-IKKβ mice lacking p16 or p19 individually and to study their sensitivity to skin cancer.

## 5. Lessons from Human Tumoral Samples

In the last years, a huge amount of genomic and expression data from numerous human tumoral samples have been generated. These data show association in some cases between tumor development and genetic or expression alterations in *IKBKB*.

For example, IKKβ expression seems to be a risk factor in ovarian cancer and higher expression of IKKβ in human ovarian cancer samples is associated to lower patient survival. In agreement with these data, pharmacological inhibition of IKKβ or downregulation by RNA interference in ovarian cancer cell lines led to a decrease in some characteristics related to tumoral aggressiveness (as anchorage-independent growth or invasion through basement membrane) [73].

*IKBKB* was recognized as a cancer gene by COSMIC based on the finding of *IKBKB* activating mutations in Lys^175^ in around 8% of splenic marginal zone lymphomas of B cells; this mutation renders a constitutively active IKKβ protein [74]. A relatively high number of mutations in *IKBKB* have been found in skin basal cell carcinomas, although the interpretation of these data is difficult due to the high mutation rate found in this particular tumor type [75]. In spite of this, in general, *IKBKB* is rarely mutated and COSMIC finds only five tumor types with mutations and always at low proportions. Lys^175^ Glu is the most frequently found mutation, although apparently there are no hotspots. In The Cancer Genome Atlas (TCGA [76,77,78,79]) there are also very few mutations affecting the *IKBKB* gene.

Gene fusions involving *IKBKB* have also been found, although this process does not seem to be a common mechanism of activation of the gene, as at the moment only a few have been found in breast [80] and prostate tumors [81].

**Figure 2:**
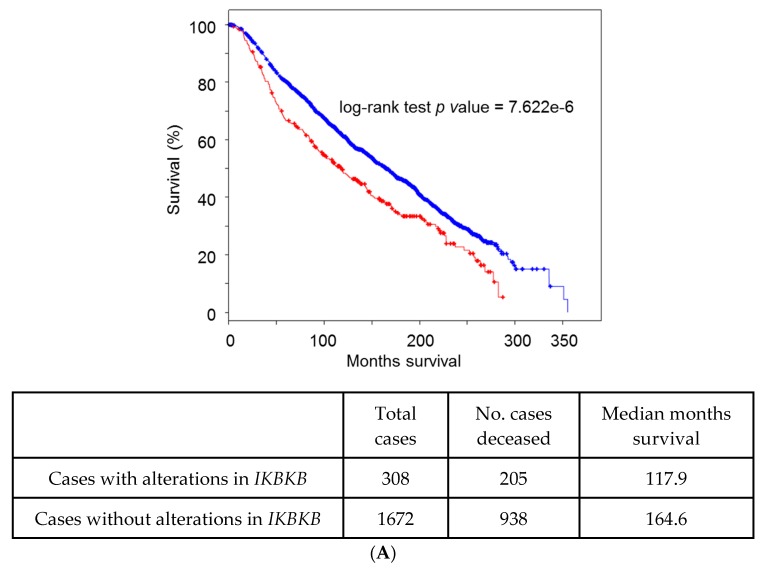

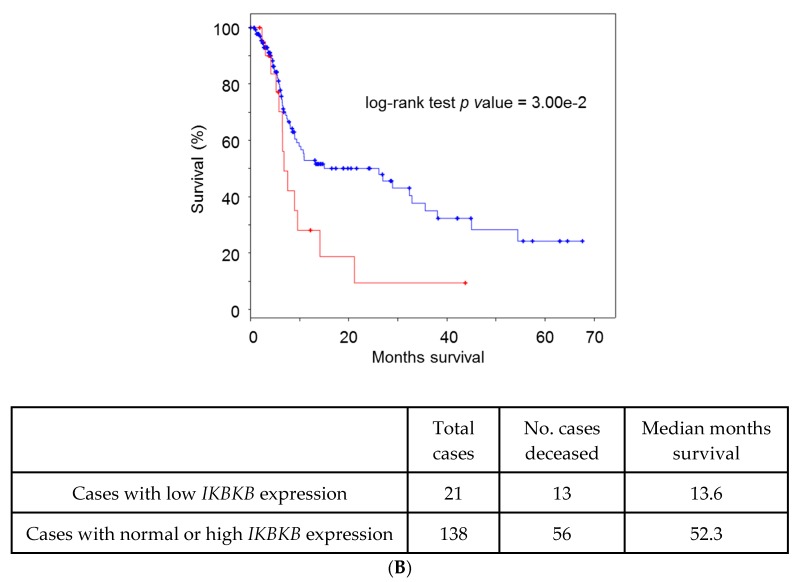
(**A**) Survival (months), in the TCGA Breast METABRIC cohort, separating tumors with homozygous or heterozygous deletion of *IKBKB* (**red**) from tumors without loss of *IKBKB* (**blue**). Tumor data and statistical and graphical tools are from cBioportal [78,79]; (**B**) Survival (months) in the TCGA Head and Neck Squamous Cell Carcinomas (HNSCC, Nature 2015 cohort), separating tumors with low *IKBKB* expression (fold change <−1.5) (**red**) from all the rest (**blue**). Tumor data and statistical and graphical tools are from cBioportal [78,79].

Homozygous deletions are also rare, being found in TCGA mainly in prostate (5% of tumors) and liver tumors (around 4%). No particular association with any tumor variable or mutation has been found. It is intriguing that some tumors show loss of one single *IKBKB* allele, in special liver and prostate tumors (40% of the tumors) and head and neck SCCs (35%), which seems to indicate a tumor suppressive role of IKKβ in these cancer types. Interestingly, heterozygous loss or low expression of *IKBKB* is associated to mutations in *TP53* and to impaired overall survival in breast tumors (Figure 2A) and in Head and Neck Squamous Cell Carcinomas (HNSCCs) (Figure 2B).

The analysis of the data included in cBioportal indicates that overexpression of *IKBKB* and copy-number amplifications are common, being *IKBKB* amplified at high level in around 25% of neuroendocrine prostate tumors. The same holds true in 10% of breast tumors and in around 5% of several other types of tumors in the uterus, ovary, esophagus, lung and bladder. In these tumor types, the possible existence of correlations between IKKβ expression and any biological or clinical variable is not known but in concordance with the previously mentioned association between low expression of *IKBKB* and mutations in *TP53* in breast cancer, overexpression of *IKBKB* in breast cancer correlates negatively with TP53 mutation but positively with ER-positive status (*p* < 0.0001, Fisher’s exact test, both for data in the METABRIC and TCGA (provisional) breast cancer cohorts).

Overall, these data add to the growing body of IKKβ protumorigenic and antitumorigenic evidence obtained using genetically modified animal models, indicating that the effect of IKKβ over tumoral transformation is cell-type specific, favoring carcinogenesis in many cell types but not in others. The knowledge of the biological determinants that define the pro- or anti-tumorigenic role of IKKβ is a challenge that remains open. Consequently, further work is needed, both in animal models and with human-derived tumoral samples, to unravel the cell-type specificity and complex activities of IKKβ in cancer.

## Figures and Tables

**Table 1:** Summary of mouse models relevant for the study of the role of IKKβ in cancer.

Type of Tumor/IKKβ Modification	Phenotypic Effect	Proposed Mechanism	References
**Lung Cancer**			
Lentiviral-mediated *Ikbkb* deletion in a lung model of adenocarcinoma expressing Kras^G12D^ and shp53.	Attenuated tumor proliferation and significantly prolonged mouse survival.	Down-regulation of the NF-κB target TIMP-1 and ERK pathway; reduced cell proliferation.	[47]
**Melanoma**			
Dox-induced *Ikbkb* deletion in melanocytes expressing HRas^G12V^ in INK4A/ARF-null background.	Inhibition of melanoma tumor development.	p53-dependent cell cycle arrest and apoptosis.	[51]
*Ikbkb* deletion in myeloid cells in a mouse model injected with BRAF^V600E^ /PTEN^−/−^ melanoma cells.	Growth of cutaneous and lung melanoma tumors.	Myeloid IKKβ promotes antitumor immunity by modulating the chemokine CCL11 and the innate immune response.	[52]
**Pancreatic Cancer**			
Cre-mediated *Ikbkb* deletion in a model expressing Kras^G12D^ in the pancreas.	Reduced progression of pancreatic neoplasia.	Downregulation of inflammatory cytokines and chemokines; downregulation of Notch signaling; PPARG inhibition.	[53]
Cre-mediated *Ikbkb* deletion in a model expressing Kras^G12D^ in the pancreas (also in INK4A/ARF null background).	Reduced formation of pancreatic neoplasia and of pancreatic ductal adenocarcinomas.	Inhibition of inflammation and NFκB activation.	[54]
**Liver Cancer**			
Cre-mediated *Ikbkb* deletion in hepatocytes.	Enhanced DEN-induced hepatocarcinogenesis.	Increased cell death and compensatory proliferation mediated by increased ROS production and JNK1 activation.	[55,56]
*Ikbkb* deletion in initiated hepatocytes transplanted onto mice expressing PLAU in hepatocytes.	Enhanced formation of hepatocellular carcinomas.	Enhanced ROS production and STAT3 activation.	[57]
Cre-mediated *Ikbkb* deletion in hepatocytes and hematopoietic-derived Kupffer cells.	Decreased DEN-induced hepatocarcinogenesis and reduced hepatocyte regeneration.	Diminished induction of hepatic mitogens (IL-6, TNFα and HGF).	[55]
**Intestinal Cancer**			
Cre-mediated *Ikbkb* deletion in intestinal epithelial cells.	Decreased tumor incidence in a colitis-associated cancer model.	Enhanced p53-independent apoptosis and defective Bcl-xL induction in tumor promotion.	[58]
Cre-mediated *Ikbkb* deletion in myeloid cells.	Decreased tumor incidence and size in a colitis-associated cancer model.	Reduced expression of proinflammatory mediators without effect on apoptosis.	[58]
Expression of constitutively active IKKβ in intestinal epithelial cells.	Spontaneous tumorigenesis and enhanced carcinogenesis induced by APC mutation or chemical treatments.	Activation of Wnt signaling and production of a pro-inflammatory intestinal microenvironment.	[59]
*Ikbkb* deletion in mesenchymal cells mediated by a constitutive ColVI-Cre transgene.	Protection against inflammation-induced intestinal carcinogenesis.	IKKβ in mecenchimal cell causes an increase in IL-6 production and STAT3 activation.	[60]
*Ikbkb* deletion in mesenchymal cells mediated by an inducible Col1a2Cre-ER transgene.	Stimulated intestinal proliferation, increased angiogenesis and promotion of colonic tumor growth.	IKKβ down-regulates TGFβ signaling and HGF secretion.	[61]
**Non-Melanoma Skin Cancer**			
Constitutive K14-Cre mediated *Ikbkb* deletion in epidermal keratinocytes.	Severe inflammatory skin disease leading to death before postnatal day 10.	Unbalanced immune skin homeostasis, mediated by TNFα.	[62]
Inducible K14-CreER mediated *Ikbkb* deletion in epidermal keratinocytes.	Skin inflammation, hair follicle disruption and epidermal pseudoepitheliomatous hyperplasia but not tumor formation.	*Ikbkb* deletion leads to STAT3 and ERK1/2 activation.	[63]
Unexpected *Ikbkb* deletion in skin keratinocytes mediated by a GFAP-Cre transgene.	Skin hyperplasia, inflammation and development of SCCs in part of the mice.	Increased TNFα expression in lesions.	[64]
Unexpected *Ikbkb* deletion in skin keratinocytes mediated by a OX40-Cre transgene.	Hyperplasia and inflammatory skin lesions.	Increased TNFα expression in lesions and T lymphocyte activation.	[65]
IKKβ overexpression in epidermal keratinocytes by a K5-IKKβ transgene.	Resistance to tumor development in chemically-induced NMSC models.	Tumor-protective function of IKKβ is mediated by tumor suppressor proteins p16 and/or p19.	[66]
**Oral and Esophageal Cancer**			
IKKβ overexpression in oral epithelial keratinocytes by a K5-IKKβ transgene.	Spontaneous oral tumoral lesions and increased malignancy after oral chemical carcinogenesis.	Enhanced oral inflammation with infiltration of granulocytes, macrophages and B lymphocytes.	[67]
Expression of constitutively active IKKβ in esophageal epithelia.	Esophagitis and increased angiogenesis in esophageal stroma.	Increased production and secretion of GM-CSF and TNF.	[68]

ROS: reactive oxygen species; SCC: squamous cell carcinoma; NMSC: non-melanoma skin cancer.
